# The Transactions of the Provincial Medical and Surgical Association. Vol. XI

**Published:** 1843-10-01

**Authors:** 


### THE TRANSACTIONS OF THE PROVINCIAL MEDICAL AND SURGICAL ASSOCIATION. Vol. XI. Churchill, 1843.

THIS volume will keep up the reputation acquired by its predecessors. Its contents are—1. The Retrospective Address, delivered at the Tenth Anniversary Meeting of the Provincial Medical and Surgical Association, held at Exeter, August 3rd and 4th, 1842. By James Black, M.D. II. On the Medical Topography of Sidmouth, being a Sketch of the Medico-Topography, Geology, Natural Productions, and Statistics of that District. By J. D. Jeffery, Esq.—III. Experimental and Practical Researches on the Structure and Function of Blood Corpuscles; on Inflammation; and on the Origin and Nature of Tubercles in the Lungs. By William Addison, F.LS.—IV. Some Cases showing the Advantage of Powerful Counter-irritation, especially the Long Issue on the Calvarium. By George Wallis, M.D.—V. On the Employment of Extension in the Treatment of Fractures of the Spine; with a case. By William Henchman Crowfoot, Esq.—VI. A Case of Paralysis of the Serratus Magnus, which caused the Lower Angles of the Right and Left Scapulæ to become disengaged from the Latissimus Dorsi, &c. By John M. Banner, Esq. VII. Remarks on Matico, a Styptic much used in South America, for the Suppression of Hæmorrhage. By Thomas Jeffreys, M.D.— VIII. Anatomico-Chirurgical Observations on Dislocations of the Astragalus. By Thomas Turner, Esq.

I. The *Retrospective Address* by Dr. Black is able and complete. Its nature precludes any notice, at present, from us.

### II. MEDICAL TOPOGRAPHY OF SIDMOUTH. By *J. D. Jeffery*, Esq.

From very extended observations on the climate of Sidmouth, Mr. Jeffery arrives at the following conclusions with regard to its applicability to different affections and diseases.

"In all cases where disease or disorder is accompanied with a *relaxed* habit of body, with softness of muscular fibre and paleness of skin; in chronic affections of the liver, in chlorosis, anæmia, *atonic* dyspepsia, in uterine disorders, arising from debility—to these this coast cannot be said to be adapted in the summer months; in the autumn and winter they may be benefitted. This observation does not apply to convalescents from any protracted acute disease, such as fever, or from accidents, who, coming from inland, will be very likely to recruit their health in the summer; neither does it apply to weakly children, for whom sea bathing may be considered desirable. I have known persons suffering from chronic irritation and catarrh of the bladder, derive comfort in this summer climate; and also those who labour under acute inflammatory dyspepsia. Those who are subject to different and *peculiar* nervous affections, and are very sensitive to cold, live comparatively comfortable here all the year round. Asthma, that form so called spasmodic, congestion of the lungs, hæmoptysis, and cases wherein there is great irritability of the pulmonary mucous membrane, will be likely to receive benefit at *all seasons* in the soft moist air of the south coast. For young and delicate persons, in whom a tendency to pulmonary disease is suspected, a residence here is desirable. Some forms of inflammatory rheumatism are benefitted. In some peculiar affections of the brain, also attended with *excitement*, this climate proves soothing; but those of a *melancholic* temperament should not be brought here, except in the autumn and winter.

"With regard to pulmonary affections, there is much discrimination to be observed. I have previously mentioned the precautions and considerations which, in my opinion, should be the necessary accompaniments to a change of air, when prescribed for the consumptive invalid. In tuberculous cachexy, and in suspected tuberculous deposit, when the circulation is quick and the lungs sensitive to cold, a *permanent* residence on this coast, subject to the necessary and paramount auxiliaries, such as a proper choice of situation, aspect, &c., and the possession of general, mental, and bodily comforts, with judicious care, might be of essential service. Where softening of tubercle in the lung has taken place, and the patient is in the last stage of consumption, I think I have seen both comfort and life prolonged by the patient's residing *close* to the sea, even in the heat of summer, through the effects of the refreshing qualities of the sea breezes. In winter the patient should reside in one of the numerous cottages inland." 195.

III. The Researches on the Blood Corpuscles, &c., by Mr. Addison are highly interesting. We shall advert to them on another occasion, and leave them, therefore, for the present.

### IV. On the Employment of Extension in the Treatment of Fractures of the Spine. By *Wm. Henchman Crowfoot*, Esq.

The narrative of a case of injury of the spine. A coachman was struck on the back of the neck, and bent double; the spinous processes of the ninth and tenth vertebræ were divided from each other considerably beyond their usual distance, the body of the ninth vertebra having been forced forward, whilst that of the tenth projected backwards. There was total loss of voluntary motion and sensation in the lower extremities.

By means of gradual extension, the deformity of the spine was diminished, and sensibility, but not motion, almost immediately restored. The patient was then placed on his back on a firm bed, and kept quiet; at the end of two months he was able to support himself on his leg, and by the end of the year, was able again to mount the box.

### V. A Case of Paralysis of the Serratus Magnus, which caused the lower Angles of the Right and Left Scapulæ to become disengaged from the Latissimus Dorsi. By *John M. Banner*, Esq.

The *gist* of the paper is so well expressed by its title that it is quite unnecessary for us to add anything.

### VI. REMARKS ON MATICO, A STYPTIC MUCH USED IN SOUTH AMERICA, FOR SUPPRESSION OF HÆMORRHAGE. By *Thomas Jeffreys*, M.D.

Two kinds of *Matico* are imported, one collected when *green*, the other when ripe and of a more *yellow* appearance; the latter is stated to be the more powerful styptic. It may be employed internally or externally. *Externally*, the under side of the leaf is preferable to the powder, and more powerful than the smooth or upper side. *Internally*, the decoction or infusion (half an ounce or an ounce to the pint), may be given in doses of three tablespoonfuls.

### VII. ANATOMICO-CHIRURGICAL OBSERVATIONS ON DISLOCATIONS OF THE ASTRAGALUS. By *Thomas Turner*, Esq.

This bone may be displaced—*forwards, forwards and inwards, forwards and outwards, upwards and outwards, outwards, inwards, backwards, and outwards, downwards and backwards.*

The dislocation may be partial or complete, simple or compound.

The signs of these accidents of course vary, but in all *complete* dislocations, simple or compound, direct or indirect, the foot will be approximated to the leg by the action of muscles; the tibia will occupy a part of the hollow which has been vacated by the astragalus, and the leg will accordingly be shortened one inch or more, corresponding to the depth of the bone that has been dislodged from the ancle joint.

In partial, as well as in complete luxations, the astragalus may be normal in position, or it may undergo a change of axis with respect to its articular surfaces. And this is a matter of some consequence, as in the most promising cases, the latter of these states of the bone would offer an almost insuperable bar to reduction. In partial dislocation, where the bones of the leg are fractured, and the ligaments torn, so as to give increased space, and more power and command over the parts, inversion or eversion, or even the turning over upon itself of the astragalus, might not be an insuperable obstacle; but in complete dislocation, whether simple or compound, or complicated, with fracture or not, and especially in the second case, reduction is, the author thinks, almost impracticable.

In dislocations *forwards*, the astragalus may be partially or wholly pushed from between the tibia and fibula and calcaneum, without lacerating the skin; but almost invariably, the pressure of the bone will cause, sooner or later, the skin to give way, and the bone to protrude.

Dislocations *inwards* and *outwards* are generally simple, the subcutaneous textures being strong enough to prevent the bone from being driven through them. In dislocations *inwards*, however, the bone has always sloughed out ultimately, whilst in dislocation *outwards*, the bone has remained in its new situation, without making its way to the surface.

Dislocation *backwards* is always simple and always remains so; and this is readily understood when we recollect the nature of the soft structures which occupy the space between the joint and the tendo-achillis. This dislocation is very rare.

Mr. Turner details 45 cases of this accident; of these there are,

*Simple cases*—24

| | | | |
|---|---|---|---|
| *Partial* .. | 3 | Without fracture .. .. | 2 |
| | | With fracture .. .. .. | 1 |
| *Complete*.. | 21 | Without fracture .. .. | 13 |
| | | With fracture .. .. .. | 8 |

*Compound cases*—21.

| | | | |
|---|---|---|---|
| *Partial* .. | 2 | Without fracture .. .. | 2 |
| | | With fracture .. .. .. | 0 |
| *Complete*.. | 19 | Without fracture .. .. | 13 |
| | | With fracture .. .. .. | 5 |
| | | With dislocation of ancle .. | 1 |

Thus, there is no great disparity in numbers between the simple and compound cases.

With regard to the direction of the dislocation:—11 were *forwards; 4 forwards and inwards;* 10 *forwards and outwards;* 1 *upwards and outwards;* 6 *inwards;* 6 *outwards;* 6 *backwards;* 1 *outwards, downwards and backwards.*

*Principles of the Treatment in Dislocations of the Astragalus.*

1. *Reduction.*—If the astragalus be only partially dislocated, and not twisted round, reduction may frequently be accomplished, because the main obstacle to success, namely the forcible approximation of the tibia to the os calcis, is prevented by the part of the astragalus which remains between these bones. If there is fracture of the leg, fracture of the os calcis, or dissevering of the bones of the tarsus with or without fracture, reduction is practicable. Supposing, however, the astragalus to be completely luxated, without fracture, disjointed tarsal bones or dislocation of the ancle, then, Mr. Turner is of opinion, that reduction is hopeless, and that all attempts at accomplishing it are worse than useless; that to the violence used, we may attribute much of the mischief which arises from diffuse cellular inflammation, from immediate gangrene induced in the parts by the direct pressure which has been applied, and other destructive consequences which increase considerably the danger of the accident.

The main obstacle to reduction consists in the rigid and unyielding approximation of the leg to the foot by the powerful action, not merely of the gastrocnemii, but of all the muscles which pass from the leg to the foot behind the malleoli: by flexing the leg upon the knee we can relax the gastrocnemius, but have no means of overcoming the other muscles, as the foot in this accident is often fixed and cannot be extended so as to enable us to put these muscles in a state of relaxation.

2. *The practice of allowing the Astragalus to remain in its new situation.*—If the astragalus cannot be reduced, the question arises as to what is the proper course to be pursued. In almost all the cases narrated in which the bone was dislocated *backwards,* the cases were left to nature, and did well; a very useful foot being the result. In other cases the following, the author thinks, the safest line of practice.

"In partial cases, whether simple or simple and complicated, should attempts at reduction fail, there must be no operative interference. In partial and compound, or compound and complicated, (reduction failing,) excision, if practicable, of the protruded portion of bone should be performed. This proceeding gives great facilities to the proper adaptation of the parts; but leaving this out of the question, by neglecting partial excision we incur one of two risks: firstly, the exposed or prominent part of the bone may die, and the patient have to undergo the tedious and trying processes of inflammation, suppuration, and exfoliation, and the death of one part of the bone may endanger the vitality or disease of the remainder, and ultimately involve the ankle joint (as in one of M. Boyer's cases), and demand, as a last resource, amputation as the means which alone could give to the patient a chance of life; and if the extruded portion of bone should not be excised, and continue to live, its presence would so far restrain the action of the ankle as to produce permanent contraction of the heel, permanent inflexibility, and permanent lameness, as in one of our recorded cases." 467.

3. *Excision of the Astragalus.*—It may be summarily stated, that in simple, direct, and complete luxation, the author advocates the practice of allowing the bone to remain in its new situation, without any operation, until it manifests a tendency to ulcerate the skin, in which case he would make an incision over the bone to relieve tension and pressure; and when the bone becomes loose he would remove it.

"In simple, indirect, and complete luxation, he would anticipate, as a matter of certainty, that the bone would die and require dislodgment; to take off tension and pressure from the angles of the displaced bone, he would at once make an incision over it, but not remove the bone, wishing to benefit by the probability that the exposure of the cavity of the joint may have an injurious effect. In complete compound luxation, whether direct or indirect, or complicated, with fracture or with dislocation of the ankle joint, he would immediately proceed to the removal of the astragalus, from believing that the limb will be put in a better condition for the reparative process of the joint, by the abstraction of the processes of inflammation, suppuration, ulceration, and sloughing (processes necessary to the disengagement of the astragalus by natural efforts); for if these be saved, nature will be able to direct, undividedly, her sanatory operations to the interior or deeper seated parts; whereas, if her powers are divided between the extrication of the astragalus from its abnormal situation, and the reparation of the joint, they might be insufficient for the purposes required, and the limb or life fall a sacrifice." 475.

4. *Amputation.*—Contrary to what might be expected, it is well known that very serious injuries may affect the ancle, without the removal of the limb being required. The injuries in connection with the present subject, which require the *immediate* amputation of the limb, are extensive laceration and contusion of soft parts, united with simple, simple and complicated, compound, or compound and complicated dislocations. The destruction of the soft parts ought to be extremely severe to render amputation necessary, but much must of course depend, in each individual case, upon the age, habits, &c. of the patient.

*Subsequent amputation* of the limb may be required at different periods after the accident; traumatic gangrene may manifest itself in the course of a few days, not unfrequently, the author imagines, in consequence of the violence exerted in the attempts at reduction. Or consecutive amputation may be required in case of deficient reparative powers, or from extensive suppuration, sloughing, &c. of the cellular and other tissues.

A very instructive paper.

